# Supplementary material for: A Global Screen for Assembly State Changes of the Mitotic Proteome by SEC-SWATH-MS
Source: Cell Syst. 2020 Feb 26;10(2):133–155.e6. doi: 10.1016/j.cels.2020.01.001 (PMC7042714; doi:10.1016/j.cels.2020.01.001)

Q92558 | WASF1_HUMAN | WASF1 KIAA0269 SCAR1 WAVE1
Monomer MW [kDa]: 61.652 Monomer expected elution fraction: 44

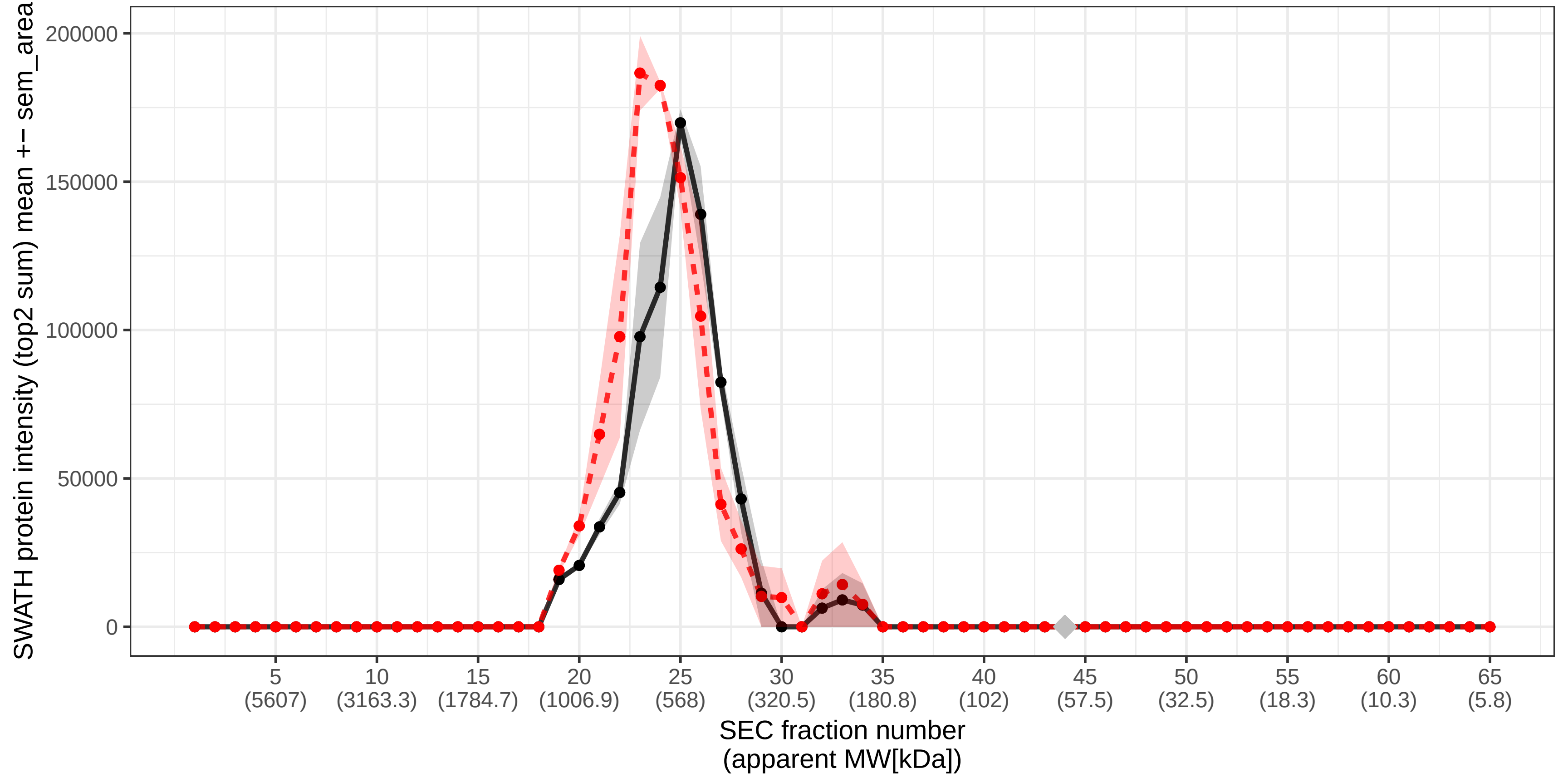

Supplement: Data S1. SEC-SWATH-MS Protein Chromatograms, Related to Figure 1 [file mmc6.zip › SECchrom_Q92558_WASF1_HUMAN_WASF1_KIAA0269_SCAR1_WAVE1.pdf]
